# Gypenoside Inhibits Bovine Viral Diarrhea Virus Replication by Interfering with Viral Attachment and Internalization and Activating Apoptosis of Infected Cells

**DOI:** 10.3390/v13091810

**Published:** 2021-09-12

**Authors:** Guanghui Yang, Jialu Zhang, Shenghua Wang, Jun Wang, Jing Wang, Yaohong Zhu, Jiufeng Wang

**Affiliations:** College of Veterinary Medicine, China Agricultural University, Beijing 100193, China; ygh564701721@163.com (G.Y.); 18404969065@163.com (J.Z.); by20173050440@cau.edu.cn (S.W.); junw123126@163.com (J.W.); wangjing100193@163.com (J.W.); zhu_yaohong@hotmail.com (Y.Z.)

**Keywords:** bovine viral diarrhea virus, gypenoside, antiviral agents, apoptosis, tight junction protein

## Abstract

Bovine viral diarrhea virus (BVDV) causes a severe threat to the cattle industry due to ineffective control measures. Gypenoside is the primary component of Gynostemma pentaphyllum, which has potential medicinal value and has been widely applied as a food additive and herbal supplement. However, little is known about the antiviral effects of gypenoside. The present study aimed to explore the antiviral activities of gypenoside against BVDV infection. The inhibitory activity of gypenoside against BVDV was assessed by using virus titration and performing Western blotting, quantitative reverse transcription PCR (RT-qPCR), and immunofluorescence assays in MDBK cells. We found that gypenoside exhibited high anti-BVDV activity by interfering with the viral attachment to and internalization in cells. The study showed that BVDV infection inhibits apoptosis of infected cells from escaping the innate defense of host cells. Our data further demonstrated that gypenoside inhibited BVDV infection by electively activating the apoptosis of BVDV-infected cells for execution, as evidenced by the regulation of the expression of the apoptosis-related protein, promotion of caspase-3 activation, and display of positive TUNEL staining; no toxicity was observed in non-infected cells. Collectively, the data identified that gypenoside exerts an anti-BVDV-infection role by inhibiting viral attachment and internalization and selectively purging virally infected cells. Therefore, our study will contribute to the development of a novel prophylactic and therapeutic strategy against BVDV infection.

## 1. Introduction

Bovine viral diarrhea virus (BVDV) is a pathogen that is distributed worldwide and is responsible for significant economic losses in the cattle industry [1]. The clinical manifestations of the virus are presented as an acute infection, fetal infection, or mucosal disease. The infection causes many direct losses, such as morbidity and mortality due to immunosuppression, reduced reproductive performance, stillbirth, growth retardation or abortion, and reduced milk production [1,2]. BVDV belongs to the genus Pestivirus of the Flaviviridae family, which includes both the border disease virus in sheep and the causative agent of classical swine fever [3,4]. The BVDV genome is a positive-sense single-stranded RNA molecule of about 12.3 kb in length [5]. It contains a unique open reading frame encoding a large polyprotein processed into 10–11 structural and non-structural proteins: N^pro^-C-E^rns^-E1-E2-p7-NS2-NS3-NS4A-NS4B-NS5A-NS5B [6]. According to the genetic and antigenic differences, BVDV can be divided into three different species: BVDV-1, BVDV-2, and BVDV-3 [7]. Meanwhile, at least 21 different genotypes of BVDV-1 have been found, while BVDV-2 only comprises four genotypes based on genetic analyses [8]. Two biotypes of BVDV are further sub-divided into two phenotypic biotypes—cytopathic (CP) and non-cytopathic (NCP)—on the basis of their capacity to induce a cytopathic effect in cell culture or not [9].

Although vaccination is one of the main approaches to the prevention of BVDV infection, a single administration cannot control or eliminate BVDV [10,11]. Wide-ranging prevention and control measures should be taken to limit BVDV infections. Thus, the exploration of effective antiviral drugs is urgent. Gynostemma pentaphyllum (Thunb.) is a traditional Chinese medicine with numerous pharmacological properties that include anti-hypertensive, anti-aging, anti-hyperlipidemia, anti-hyperglycemia, and anti-inflammatory effects [12,13]. Gypenoside, the principal ingredient of Gynostemma pentaphyllum, is effective in reducing blood parameters in the case of fatty liver disease, as well as in reducing fibrosis, improving the intestinal microbiota in non-alcoholic fatty liver disease, and protecting against its progression [14]. Gypenoside also possessed antiviral, immune-enhancing, and antioxidant efficacies. [15]. However, few studies have investigated the effects of its antiviral activity.

In the development of the body and maintenance of homeostasis, apoptosis is the main form of programmed cell death. During a microbial infection, the host defends and clears infected cells through apoptotic cell death [16]. The apoptosis of the infected cells can be an additional mechanism for resisting the infectiousness of the virus and preventing it from spreading more widely in the host [17].

In this study, we reveal the antiviral activity of gypenoside against BVDV infection. In order to clarify the antiviral mechanism of gypenoside, we further prove, for the first time, that this effect depends on the ability of its compound to activate BVDV-inhibited apoptosis in infected cells. These findings suggest that gypenoside may represent a novel therapeutic option for regulating host defense in order to overcome drug-resistant BVDV.

## 2. Materials and Methods

### 2.1. Virus, Cells, and Reagents

Madin–Darby Bovine Kidney (MDBK) cells were cultured in Dulbecco’s Modified Eagle Medium/Ham’s F-12 medium (1:1) (Gibco, Grand Island, NY, USA) supplemented with 7% fetal bovine serum (Thermo Scientific, Waltham, MA, USA) at 37 °C in a humidified atmosphere containing 5% CO_2_.

The NCP-BVDV-BJ175170 isolate strain, which was isolated from blood samples from cows with suspected BVDV infections, was maintained in our laboratory. The BVDV BJ175170 and BJ1305 isolate strains were utilized for all experiments and are represented by “BVDV” in this article.

Gypenoside (purity > 99%) was purchased from Selleck (Houston, TX, USA). Z-DEVD-FMK and ABT-263 (Beyotime Biotechnology, Shanghai, China), anti-Bcl-2 antibody and anti-Bax antibody, rabbit anti-Claudin-1 polyclonal antibody, rabbit anti-Occludin polyclonal antibody, rabbit anti-CD46 polyclonal antibody, anti-GAPDH antibody (ProteinTech Group, Rosemont, IL, USA), anti-caspase3 antibody (Cell Signaling Technology, Danvers, MA, USA), and anti-BVDV E2-specific mouse monoclonal antibody (VMRD, Pullman, WA, USA) were used in the study.

### 2.2. Drug Treatment

Gypenoside was diluted to 150 mM in DMSO and further diluted to various serially diluted compounds in PBS (final concentrations were 25, 50, 100, and 150 μM). MDBK cells were treated with gypenoside, Z-DEVD-FMK, or ABT-263 for 1 h by directly adding the diluted compound to the media before infection (or as indicated), and compounds were maintained on the cells throughout infection. The DMSO group was the infection control group, in which BVDV-infected cells were treated with DMSO. The negative control group was untreated. The replication of the virus was detected to assess the antiviral activity of the drug in the infected cells.

### 2.3. Cell Viability Assay 

MDBK cells were plated in a 96-well culture plate at approximately 4 × 104. The cells were incubated for 24 h at 37 °C in DMEM/F-12 containing 7% FBS growth medium. The concentration gradient of gypenoside was added to the culture medium for 24, 48, and 72 h. Cell viability was tested with a Cell Counting Kit-8 (CCK-8) (Beyotime Biotechnology, Shanghai, China) according to the manufacturer’s instructions. 

### 2.4. Immunofluorescence and TUNEL Staining 

The effect of gypenoside on MDBK cells infected with BVDV was detected with an immunofluorescence assay (IFA). The cells were incubated with 4% paraformaldehyde on ice for 15 min. Then, they were washed in PBS and incubated in 0.1% Triton X-100 for permeabilization. Anti-BVDV E2-protein mAb (1:1000) diluted in BSA (1%) was added to the cells for 1.5 h at 37 °C. After washing with PBS three times, cells were then incubated with Alexa Fluor 555-conjugated goat anti-mouse IgG (H–L) (1:500, Beyotime, Shanghai, China) for 1 h at 37 ℃. After washing in PBS three times, the nuclei were stained with 4’, 6-diamidino-2-phenylindole (DAPI, 100 ng/mL) for 5 min. Immunofluorescence was observed under a Nikon Eclipse Ti-U inverted fluorescence microscope (Nikon, Tokyo, Japan).

Compatibly with the above-described experimental design, MDBK cells were treated after confluence reached 80%. According to the manufacturer’s instructions, cell apoptosis was detected with a TUNEL BrightGreen Apoptosis Detection Kit (vazyme, Nanjing, China).

### 2.5. Western Blotting 

Proteins extracted from MDBK cells were resolved with 10% sodium dodecyl sulfate-polyacrylamide gel electrophoresis (SDS-PAGE). Then, they were transferred to a polyvinylidene fluoride (PVDF) membrane. After washing with PBST three times, membranes were incubated with 5% skimmed milk for 1.5 h at 37 °C and then with the following antibodies: anti-BVDV E2-protein (1:1000), anti-GAPDH (1:5000), anti-Bcl-2 (1:2000), anti-caspase3 (1:1000), anti-Claudin-1 polyclonal (1:1000), rabbit anti-CD46 polyclonal antibody (1:1000), rabbit anti-Occludin polyclonal antibody (1:1000), and rabbit anti-Bax (1:5000) overnight at 4 °C. The membranes were incubated with horseradish-peroxidase-conjugated goat anti-mouse and anti-rabbit IgG secondary antibodies (1:5000; Proteintech, Rosemont, IL, USA). The immunoreactivity was detected using Chemistar High-sig ECL Western blotting substrate (Tanon, Shanghai, China) and visualized on a Tanon 5200 system (Tanon, Shanghai, China).

### 2.6. RNA Extraction and RT-qPCR

The total RNA was extracted from MDBK cells with the an RNAiso Plus (Takara, Kyoto, Japan) and reverse transcribed into cDNA using a PrimeScript™ RT reagent Kit with a gDNA Eraser (Takara, Kyoto, Japan) according to the manufacturer’s instructions. RT-qPCR was performed to determine the viral load in ABI 7500 (Applied Biosystems, Foster City, CA, USA). Each reaction mixture of cDNA was 100 ng, and the concentration of each primer was 0.5 μM. The reaction procedure was 95 °C for 30 s, followed by 95 °C denaturation for 15 s, 62 °C annealing for 30 s, and 72 °C extension for 30 s, and the number of cycles was 30–40, followed by 95 °C for 15 s, 60 °C for 60 s, and 95 °C for 15 s. The following primers were used: BVDV 5′UTR (Forward primer 5’—TAGTCGTCAGTGGTTCGACGCC—3’, Reverse primer 5’—CCTCTGCAGCACCCTATCAG—3’); GAPDH (Forward primer 5′—AAAGTGGACATCGTCGCCAT—3′, Reverse primer 5′—CCGTTCTCTGCCTTGACTGT—3′). The CT values of the target genes in the gene expression were normalized to GAPDH and relative to the mock-infected control. Sampling was performed in triplicate in each reaction, and the data were calculated as the fold-change using the 2^−ΔΔCT^ method.

### 2.7. Viral Titration

The cells were infected with 10-fold serial dilutions of BVDV (MOI = 1.0) samples in four replicates after the confluence reached approximately 80% in the 96-well plates. The culture medium was replaced with DMEM/F-12 containing 2% FBS after 1 h at 37 °C. The plates were incubated for 48 h at 37 °C, and were then assessed with IFA. Immunofluorescence was observed using a Nikon Eclipse Ti-U inverted fluorescence microscope. BVDV titers were calculated using the Reed–Muench method.

### 2.8. Viral Attachment, Internalization, Replication, and Release Assay 

Viral inactivation assay: The mixture of BVDV (MOI = 0.5) combined with gypenoside (50 μM) or DMSO was incubated at 37 °C for 2 h. The mixture was washed with PBS and ultracentrifuged at 90,000× *g* for 1.5 h at 4 °C in 20% sucrose buffer (*w*/*w*) to purify the virus; then, the virus particles were resuspended in the culture medium prior to incubating cells at 37 °C for 2 h. Cell lysates were harvested for RT-qPCR and Western blotting analysis. 

Viral attachment assay: The cells were pretreated with gypenoside (50 μM) or DMSO for 1 h. The cells were incubated with BVDV (MOI = 0.5) at 4 °C for 2 h. After washing three times with PBS, cell lysates were harvested for RT-qPCR and Western blotting analysis. 

Viral internalization assay: To allow viral attachment, the cells were incubated with BVDV (MOI = 0.5) for 1 h at 4 °C. After washing three times with PBS to remove the unbound viruses, the cells were incubated with DMEM/F-12 2% FBS containing 50 μM gypenoside or DMSO for 1 h at 37 °C. Cell lysates were harvested for RT-qPCR and Western blotting analysis. 

Viral replication assay: The cells were infected with BVDV (MOI = 0.5) for 1 h at 37 °C. To remove non-internalized viruses, they were washed with PBS (pH 3.0). Cells were treated with DMEM/F-12 to remove the remaining PBS (pH 3.0). Then, the cells were incubated with DMEM/F-12 2% FBS containing 50 μM gypenoside or DMSO for 2 h at 37 °C. Cell lysates were harvested for RT-qPCR and Western blotting analysis. 

Viral release assay: The cells were infected with BVDV (MOI = 0.5) for 10 h at 37 °C. They were washed three times with PBS, and the cells were incubated with DMEM/F-12 2% FBS containing gypenoside (50 μM) or DMSO at 37 °C for 12 h. The supernatants were harvested for RT-qPCR and Western blotting analysis.

### 2.9. Flow Cytometry

Compatibly with the above-described experimental design, MDBK cells were treated with the agent after confluence reached 80%. Cell apoptosis was detected with an Annexin V-PE/7-AAD Apoptosis Detection Kit (vazyme, Nanjing, China) according to the manufacturer’s instructions and was analyzed through flow cytometry (BD Biosciences, San Jose, CA, USA). 

### 2.10. Statistical Analysis

Data are shown as mean ± standard deviation and were analyzed using SPSS (Statistical Package for the Social Sciences, version 17.0, Chicago, IL, USA). The statistical significance of differences amongst the mean values of multiple groups was evaluated with a one-way analysis of variance (ANOVA) or a two-tailed Student *t*-test. *p*-value < 0.05, *p*-value < 0.01, and *p*-value < 0.001 were considered statistically significant.

## 3. Results

### 3.1. Gypenoside Inhibits BVDV Replication in MDBK Cells

**Figure 1:**
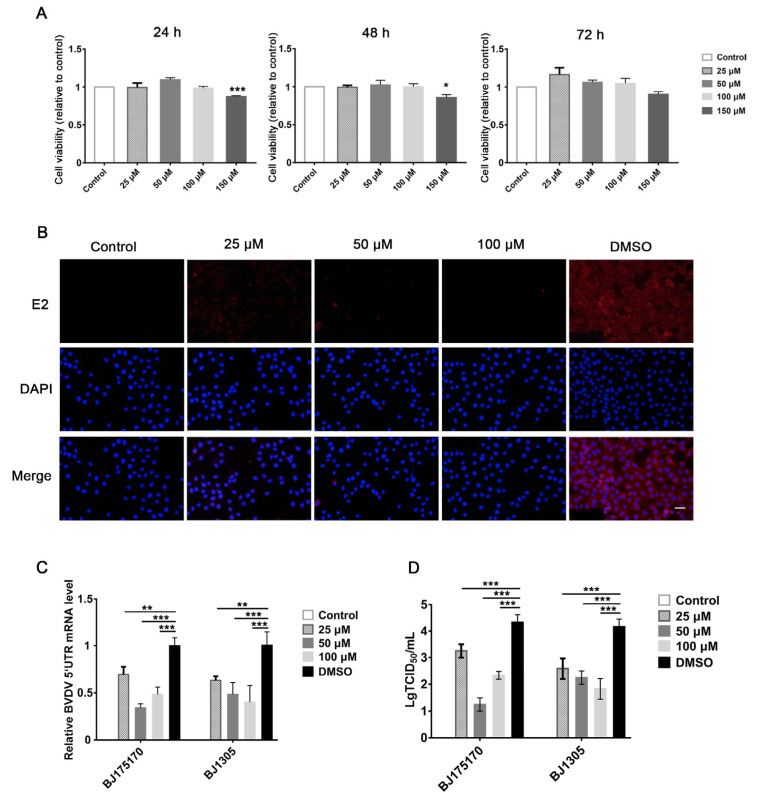
Gypenoside inhibits BVDV replication in MDBK cells. (**A**) Cell viability in MDBK cells after treatment with 25–150 μM gypenoside for 24–72 h. (**B**) IFA of BVDV-infected cells treated with escalating doses of gypenoside (25–100 μM) or DMSO. BVDV E2 protein (red) and DAPI (blue). Scale bars represent 100 μm. (**C**) The expression of BVDV 5’UTR mRNA in BVDV-infected MDBK cells treated with the indicated concentrations of gypenoside at 24 hpi. (**D**) The TCID50 result for BVDV titers in BVDV-infected MDBK cells treated with the indicated concentrations of gypenoside at 24 hpi. The control is the untreated and uninfected group. Data from three independent experiments and error bars are presented as the mean ± SEM. * *p* < 0.05, ** *p* < 0.01, *** *p* < 0.001.

**Figure 2:**
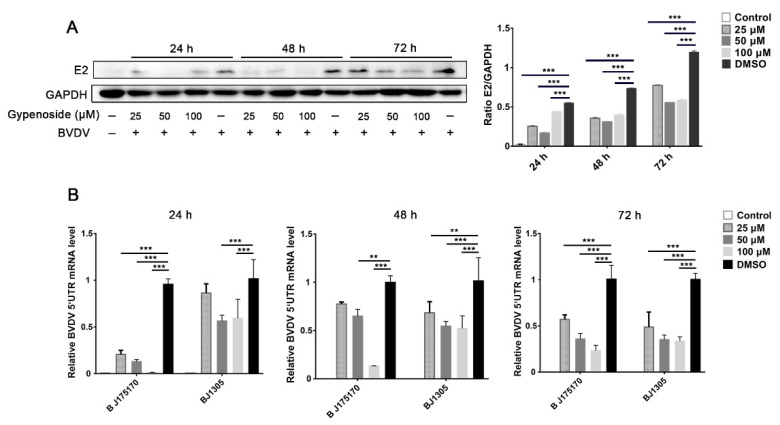
Continuous gypenoside treatment inhibits BVDV infection in the MDBK cells at 72 hpi. (**A**) The effects of the indicated concentrations of gypenoside (25, 50, and 100 μM) on BVDV infection in MDBK cells for the different times (24–72 h) detected with Western blotting. The BVDV E2 protein band was quantified as the ratio of the intensity of the BVDV E2 protein band to that of the GAPDH band in Western blotting. (**B**) The effects of the indicated concentrations of gypenoside (25, 50, and 100 μM) on the expression of BVDV 5’UTR mRNA in MDBK cells for the different times (24–72 h) detected with RT-qPCR. Data from three independent experiments and error bars are presented as the mean ± SEM. ** *p* < 0.01, *** *p* < 0.001.

To measure cell viability, MDBK cells were treated with different concentrations of gypenoside (25, 50, 100, and 150 μM) for 24, 48, and 72 h. CCK-8 assays indicated that MDBK cells treated with different concentrations of gypenoside (25, 50, and 100 μM) showed a similar cell viability to that of the untreated control (Figure 1A). Thus, these concentrations of gypenoside (25, 50, and 100 μM) were selected to follow all assays. In order to confirm the antiviral effect of gypenoside treatment, MDBK cells were treated with escalating concentrations of gypenoside for 1 h before being infected with the BVDV BJ175170 and BJ1305 isolate strains at up to 24 hpi. The number of infected cells determined with IFA decreased in the gypenoside-treated group compared with that in the DMSO-treated group (Figure 1B). The TCID50 and RT-qPCR assays showed that the gypenoside treatment caused a convincing reduction in BVDV 5’UTR mRNA levels and virus titers (Figure 1C,D). To further determine whether enduring administration of gypenoside inhibited infections with the BVDV BJ175170 and BJ1305 isolate strains at 24, 48, and 72 hpi, Western blotting and RT-qPCR assays were performed to detect the BVDV E2 protein and 5’UTR mRNA levels. Gypenoside treatment resulted in a significant reduction in the BVDV E2 protein and mRNA levels (Figure 2A,B). Thus, gypenoside reduced the accumulation of BVDV RNA and protein and infectious progeny titers in cell culture. The results described above demonstrated that gypenoside inhibited BVDV replication in MDBK cells.

### 3.2. Gypenoside Inhibits BVDV Replication by Affecting the Viral Attachment and Internalization Stage

**Figure 3:**
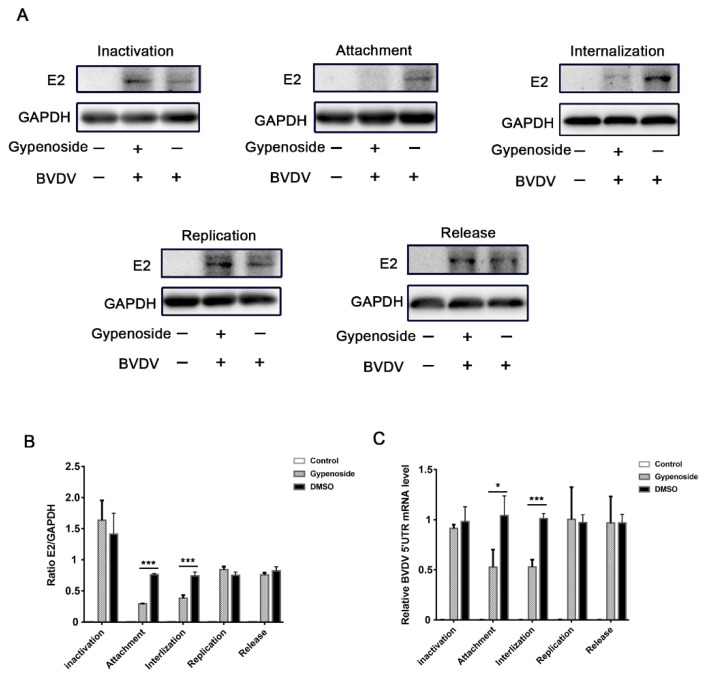
Gypenoside inhibits BVDV by affecting the viral attachment and internalization stage. (**A**) Western blotting analysis of the viral inactivation, viral binding, viral internalization, viral replication, and viral release assays in BVDV-infected MDBK cells treated with gypenoside. (**B**) The protein bands were quantified as the ratio of the intensity of the BVDV E2 protein band to that of the GAPDH band in Western blotting. (**C**) RT-qPCR analysis of the viral inactivation, viral binding, viral internalization, viral replication, and viral release assays in BVDV-infected MDBK cells treated with gypenoside. Data from three independent experiments and error bars are presented as the mean ± SEM. * *p* < 0.05, *** *p* < 0.001.

To further explore the stage when gypenoside inhibits the BVDV replication cycle, an experiment with a different pattern was performed on the BVDV-infected MDBK cells treated with a 50 μM concentration of gypenoside, which were assessed through RT-qPCR and Western blotting analysis. Firstly, we explore if gypenoside is directly antiviral and kills BVDV surviving particles. As shown in Figure 3A,C, gypenoside treatment did not directly impact the inactivated BVDV particles. Next, we further evaluated the effects of gypenoside on BVDV attachment, internalization, and replication, as shown in Figure 3A,C. The results showed that the amounts of BVDV E2 protein and RNA in BVDV-infected cells were decreased in the viral attachment and internalization stage when they were treated with gypenoside. During the viral replication and release stage, the results determined that in the BVDV-infected cells treated with gypenoside, there was no obvious inhibitory effect on the amounts of BVDV E2 protein and RNA. These results suggested that gypenoside primarily inhibited the viral attachment and internalization stage in the early steps of the BVDV replication cycle.

### 3.3. Gypenoside Downregulates Tight Junction Protein Expression in BVDV-Infected Cells

**Figure 4:**
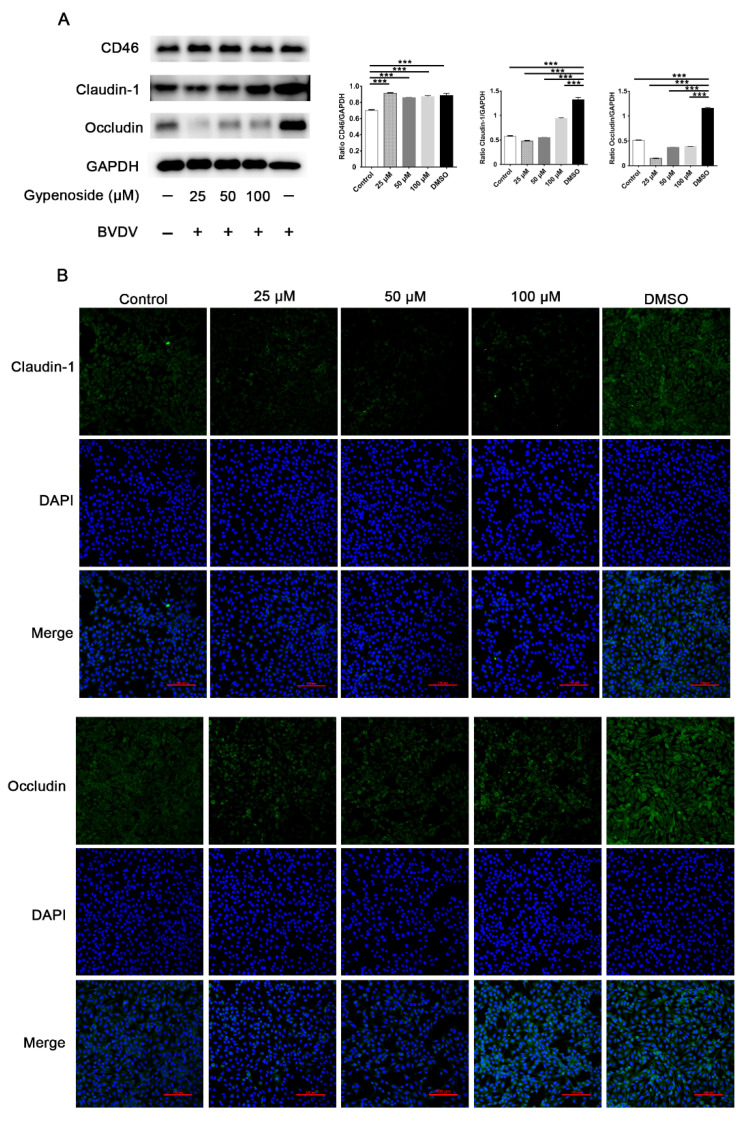
Gypenoside downregulated tight junction protein expression in BVDV-infected cells. (**A**) Representative panels showing the expression of claudin-1, occludin, CD46, and GAPDH in BVDV-infected cells treated with the indicated concentrations of gypenoside. The results are presented as the ratio of the protein band intensities of claudin 1, occludin, and CD46 to the intensity of the GAPDH band. (**B**) IFA staining for assessing the effects of the treatment with the indicated concentrations of gypenoside on claudin-1 and occludin in BVDV-infected cells. Claudin 1 or occludin (green) and DAPI (blue). Data are presented as the mean ± SEM of three independent experiments. *** *p* < 0.001.

The results for the CD46, tight junction protein claudin 1, and occludin expression in MDBK cells are shown in Figure 4. The results showed that, compared to the uninfected control, BVDV infection upregulated the expression of claudin 1 and the occludin protein (Figure 4A). Gypenoside treatment had a markedly inhibitory effect on the expression of claudin 1 and occludin protein in BVDV-infected MDBK cells compared with the DMSO group as shown through Western blotting and IFA, whereas the expression of CD46 protein, which acts as a cellular receptor for BVDV, was not different in the gypenoside groups compared with the DMSO group (Figure 4A,B). These results suggest that gypenoside downregulates tight junction protein claudin 1 and occludin expression in BVDV-infected cells.

### 3.4. Gypenoside Promotes BVDV-Inhibited Apoptosis in MDBK Cells

**Figure 5:**
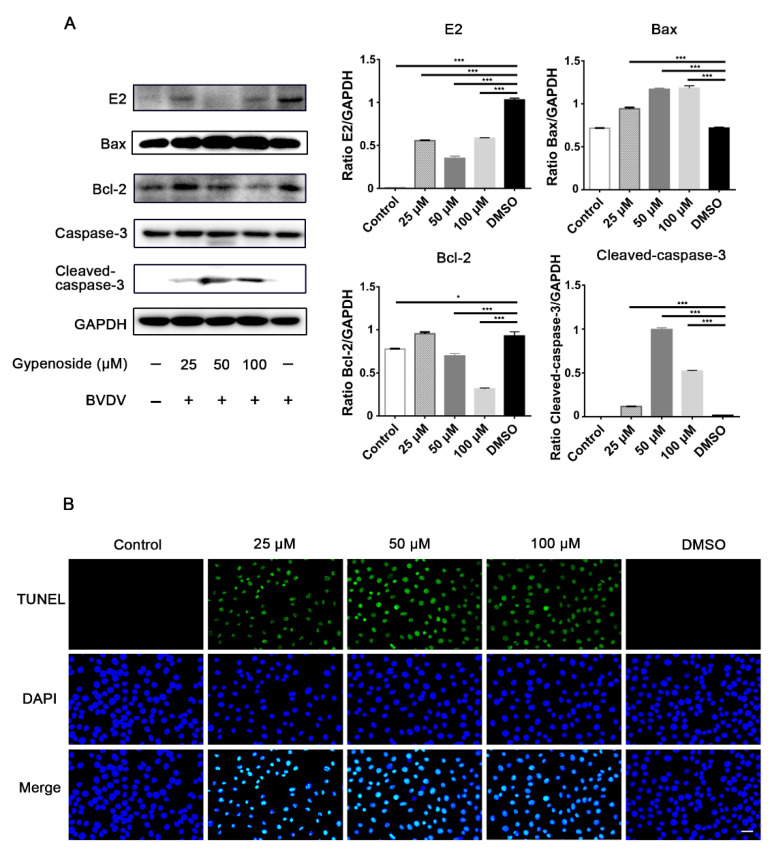
Gypenoside promotes BVDV-inhibited apoptosis in MDBK cells. (**A**) Western blotting analysis of BVDV E2, Bax, Bcl-2, and cleaved caspase-3 proteins in BVDV-infected MDBK cells treated with the indicated concentrations of gypenoside at 24 hpi. The protein bands were quantified as the ratio of the intensity of the protein bands to that of the GAPDH band in Western blotting. (**B**) TUNEL staining to assess the effects of the indicated concentrations of gypenoside on apoptosis in BVDV-infected MDBK cells at 24 hpi. TUNEL (green) and DAPI (blue). Scale bars represent 100 μm. The merging represents the co-localization of green and blue staining. Data from three independent experiments and error bars are presented as the mean ± SEM. * *p* < 0.05, *** *p* < 0.001.

BVDV-infected cells increased Bcl-2 and decreased cleaved caspase-3 protein expression compared with uninfected cells, as shown by the negative TUNEL staining, indicating that apoptosis was inhibited in BVDV-infected cells. Gypenoside treatment reduced BVDV E2 protein expression in infected cells compared with the DMSO group (Figure 5). Meanwhile, gypenoside treatment upregulated Bax and cleaved caspase-3 protein expression and downregulated the expression of Bcl-2 protein in BVDV-infected cells compared to the DMSO group (Figure 5A). Furthermore, we investigated the possibility that gypenoside activated apoptosis in BVDV-infected cells. Cells were treated with gypenoside before infection with BVDV and subsequently subjected to TUNEL assays. The results showed that TUNEL-positive cells were present in group with pretreatment with gypenoside and were significantly increased compared with those in the DMSO group (Figure 5B). Overall, these results show that gypenoside activated the BVDV-inhibited apoptosis of infected cells.

### 3.5. Apoptosis Might Be Involved in the Anti-BVDV Mechanisms of Gypenoside in MDBK Cells

**Figure 6:**
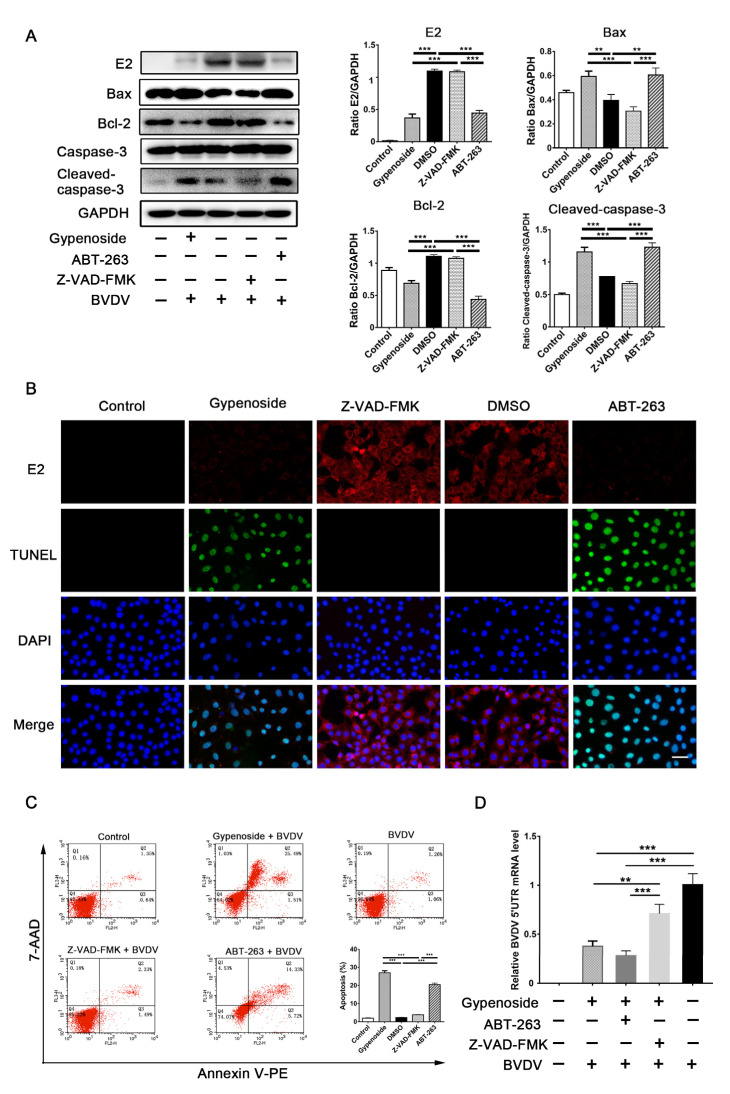
Apoptosis might be involved in the anti-BVDV mechanisms of gypenoside in MDBK cells. (**A**) Western blotting analysis of the BVDV E2, Bax, Bcl-2, and cleaved caspase-3 proteins in BVDV-infected MDBK cells treated with gypenoside, Z-DEVD-FMK, or ABT-263 at 24 hpi. The protein bands were quantified as the ratio of the intensity of the protein bands to that of the GAPDH band in Western blotting. (**B**) TUNEL and IFA staining were used to assess the effects of the treatments with gypenoside, Z-VAD-FMK, or ABT-263 on BVDV infections and apoptosis in MDBK cells at 24 hpi. BVDV E2 protein (red), TUNEL (green), and DAPI (blue). Scale bars represent 50 μm. The merging represents the co-localization of red, green, and blue staining. (**C**) Flow cytometry analysis of apoptosis in BVDV-infected MDBK cells treated with gypenoside, Z-VAD-FMK, or ABT-263 at 24 hpi. (**D**) The expression of BVDV 5’UTR mRNA in BVDV-infected MDBK cells treated with Z-DEVD-FMK or ABT-263 in the presence of gypenoside at 24 hpi. Data from three independent experiments and error bars are presented as the mean ± SEM. ** *p* < 0.01, *** *p* < 0.001.

ABT-263, a B-cell lymphoma-2 homology 3 (BH3) mimetic, can effectively inhibit the anti-apoptotic Bcl-2 protein’s activity from inducing apoptosis [18]. In contrast, Z-VAD-FMK is a pan-caspase inhibitor that can effectively block apoptosis. Therefore, Z-VAD-FMK was applied to block the transformation of caspases into apoptosis-related proteins in this study. MDBK cells were treated with ABT-263 (3 μM), Z-VAD-FMK (5 μM), gypenoside (50 μM), or DMSO before infection with BVDV, and then BVDV E2 and apoptosis-related proteins were detected through Western blotting. ABT-263 and gypenoside decreased the expressions of BVDV E2 and Bcl-2 protein and increased the expressions of Bax and cleaved caspase-3 protein in infected cells. However, Z-VAD-FMK did not affect the expressions of these proteins (Figure 6A).

Meanwhile, the TUNEL and immunofluorescence co-staining analyses showed that ABT-263 and gypenoside increased the numbers of TUNEL-positive cells and decreased the numbers of BVDV-positive cells compared with those in the Z-VAD-FMK and DMSO groups (Figure 6B). Finally, the effects of ABT-263, Z-VAD-FMK, and gypenoside on the apoptosis of infected cells were detected through flow cytometry. The results showed that ABT-263 and gypenoside significantly increased the apoptosis of infected cells, and no significant differences were found between the Z-VAD-FMK and DMSO groups (Figure 6C). The results detected through TR-qPCR showed that the BVDV 5’UTR mRNA levels were increased in infected cells treated with Z-VAD-FMK in the presence of gypenoside compared with the treatment with gypenoside alone (Figure 6D). These results indicate that apoptosis might be involved in the anti-BVDV mechanisms of gypenoside in MDBK cells. 

## 4. Discussion

BVDV is the pathogen responsible for bovine viral diarrhea (BVD) and was initially described in 1957 [19]. The NCP biotype of BVDV mainly has an impact on the host’s defenses by suppressing various aspects of the innate immune system and causing immunotolerance in the fetus for the period of primary gestation [20]. Although vaccines are the primary prevention and control measures for BVDV, they also have certain disadvantages [21]. If persistently infected (PI) cattle are administered a live vaccine contaminated with CP BVDV, they may, unfortunately, develop mucosal disease (MD) due to these co-infections [22]. Therefore, it is necessary to develop new drugs to prevent and control BVDV. Uniquely among all traditional medicines, traditional Chinese medicines (TCMs) effectively alleviate complex diseases through a multi-target/multi-component approach [23]. It has been reported that gypenoside is a significant component of the TCMs in Asia and has therapeutic effects on several diseases [24,25]. Here, we described that the compound gypenoside, a component of Gynostemma, had an inhibitory effect on the replication of BVDV in MDBK cells. In addition, viruses mainly reproduce progeny viruses by binding to cell receptors, entering cells, replicating, and releasing them. This process provides many opportunities for the development of antiviral strategies [26]. 

The growth curves of BVDV showed that within 12 hpi, the number of viruses remained stable, followed by a rapid increase in the viral load in the cell supernatant. The logarithmic growth phase of BVDV is between 12 and 48 h after inoculation. After 48 h of exposure, BVDV is basically in a stable period. These results showed that gypenoside could inhibit BVDV infection in MDBK cells from 24 to 72 hpi, indicating that gypenoside suppressed BVDV replication not only at the initial growth phase, but also at its logarithmic growth phase. To further explore the impact of gypenoside on the replication cycle of BVDV, the replication of BVDV-infected MDBK cells that were treated with gypenoside was measured. In order to confirm the anti-BVDV activity of gypenoside during the release of BVDV, the viral replication of BVDV-infected MDBK cells was examined, and it was not effective in the presence of gypenoside after 10 hpi. Gypenoside treatment inhibited BVDV replication in the viral attachment and internalization stages. The results showed that gypenoside played a role in inhibiting attachment to and internalization in of BVDV-infected MDBK cells.

It has been reported that bovine CD46 acts as a cellular receptor for BVDV. An increased susceptibility of porcine cells expressing a combination of bovine CD46 to BVDV was shown [27]. To confirm the inhibitory effect of gypenoside on the expression of CD46 with respect to the attachment to and internalization in of BVDV-infected MDBK cells, we measured the expression of CD46 with a Western blot. We found that the expression of CD46 did not change in BVDV-infected cells that were treated with gypenoside. However, it is necessary to further verify if gypenoside inhibits the plasma membrane targeting of CD46 without affecting the level of total CD46. Interestingly, the protein expression of claudin 1 and occludin was increased in the BVDV-infected cells, but was decreased in the BVDV-infected cells treated with the indicated concentrations of gypenoside. The expression of the claudin 1 protein was markedly increased in the MDBK cells after the BVDV infection [28]. Claudin 1 and occludin are considered to be essential hepatitis C virus receptors or coreceptors in infected cells [29]. Our findings suggest that gypenoside may inhibit the attachment and internalization of BVDV to MDBK cells by down-regulating the tight junction expression in the cells and that claudin 1 and occludin may be important factors for the cell entry of BVDV.

In order to establish a persistent infection, a virus must overcome the defense mechanisms of both the innate and the specific immune responses [30]. Apoptosis is a defense mechanism that is strictly regulated in the organism and is induced in many different ways. Organisms can limit the replication and spread of viruses through this mechanism. Therefore, many viruses have evolved strategies for disrupting the apoptosis mechanism through constant host–pathogen interactions and by interfering with one or more apoptosis-inducing pathways. Several DNA viruses, such as the rubella virus, have been reported to attenuate the formation of pores in mitochondria through capsid proteins, thereby hindering the release of cytochrome C and the downstream activation of caspase 3 [31,32,33]. Another example is the Epstein Barr virus, which encodes a viral homologue of Bcl-2 [32]. Not only that, but some viruses can also trigger cells to produce Bcl-2. Hepatitis C virus and human T-cell leukemia virus (HTLV) both induce Bcl-XL expression, and HTLV also induces Bcl-2 expression by involving the activation of NFκB [34,35,36]. In line with this, we observed increased activation of Bcl-2 in BVDV-infected cells, suggesting that it is involved in the observed induction of Bcl-2. The previous study demonstrated that poly(I:C) or a synthetic double-stranded RNA (dsRNA)-induced apoptosis and the expression of interferon (IFN) were inhibited in NCP BVDV-infected cells [36]. The results of this study showed that in BVDV-infected cells, not only was the expression of the apoptosis-related protein caspase 3 inhibited, as shown by Western blotting but the TUNEL staining was also shown to be negative, indicating that the NCP BVDV inhibited apoptosis in infected cells. Specifically, NCP BVDV can inhibit cell death induced by dsRNA and polyadenyluridine in infected cells. However, staurosporine- and actinomycin-D-induced cell death was not inhibited by NCP BVDV [37]. The CCK-8 results initially showed that gypenoside had no toxicity in uninfected cells. In addition, we found that in BVDV-infected cells that were treated with gypenoside, the expression of the apoptosis-related protein cleaved caspase-3 significantly increased, and the TUNEL staining was positive, implying that gypenoside inhibited the replication of BVDV by selectively activating apoptosis in BVDV-infected cells.

An orderly cell death process, apoptosis, is used by multicellular organisms to remove damaged, abnormal, or infected cells. Apoptosis is an antiviral process in cells and can be exploited to develop effective antiviral drugs [38]. Small molecules that induce the death of infected cells without affecting uninfected cells limit the replication and spread of viruses, which favorably supports the potentially broad antiviral function of apoptosis [39]. We observed decreased Bcl-2 expression and increased cleaved caspase-3 expression with Western blotting and increased apoptotic cells with a TUNEL assay and flow cytometry in infected cells that were treated with gypenoside or ABT-263. The results demonstrated that gypenoside and the apoptosis-inducing molecule ABT-263 could selectively activate apoptosis in BVDV-infected cells. Moreover, the apoptosis inhibitor Z-VAD-FMK did not affect the apoptosis of infected cells and could not inhibit the replication of BVDV. Treatment with Z-VAD-FMK could attenuate the antiviral effects of gypenoside. These results clarified, for the first time, that activating apoptosis in BVDV-infected cells contributes to the inhibitory role that gypenoside exhibits in BVDV replication. 

Altogether, the present results indicate that gypenoside can effectively inhibit the replication of BVDV in MDBK cells. Further research has confirmed that the compound is targeted toward the inhibition of the attachment and internalization stages of the BVDV replication cycle, thereby affecting viral infections in MDBK cells. The mechanism of gypenoside against BVDV is mainly through the selective activation of the BVDV-inhibited apoptosis of infected cells. This finding proves that gypenoside has the potential to become a new and effective antiviral drug against BVDV infection.

## Data Availability

Not applicable.

## Abbreviations

Bcl-2: B-cell lymphoma-2; Bax: BCL2-associated X protein; BSA: bovine serum albumin; DMSO: dimethyl sulfoxide; mAB: monoclonal antibody; MOI: multiplicity of infection; PBS: phosphate-buffered saline; TCID50: tissue culture infectious dose 50; TUNEL: terminal deoxynucleotidyl transferase-mediated dUTP-biotin nick end labeling; UTR: untranslated regions.
